# Leptin Signaling in the Ovary of Diet-Induced Obese Mice Regulates Activation of NOD-Like Receptor Protein 3 Inflammasome

**DOI:** 10.3389/fcell.2021.738731

**Published:** 2021-11-03

**Authors:** Marek Adamowski, Karolina Wołodko, Joana Oliveira, Juan Castillo-Fernandez, Daniel Murta, Gavin Kelsey, António M. Galvão

**Affiliations:** ^1^Department of Reproductive Immunology and Pathology, Institute of Animal Reproduction and Food Research of Polish Academy of Sciences, Olsztyn, Poland; ^2^Centro de Investigação em Ciências Veterinárias, Lusófona University, Lisbon, Portugal; ^3^Epigenetics Programme, The Babraham Institute, Cambridge, United Kingdom; ^4^Centro de Investigação Interdisciplinar Egas Moniz (CiiEM), Escola Superior de Saúde Egas Moniz, Campus Universitário, Monte de Caparica, Portugal; ^5^Centro de Investigação Interdisciplinar em Sanidade Animal (C.I.I.S.A.), Faculty of Veterinary Medicine, University of Lisbon, Lisbon, Portugal; ^6^Centre for Trophoblast Research, University of Cambridge, Cambridge, United Kingdom

**Keywords:** ovary, inflammation, obesity, NLRP3 inflammasome, leptin

## Abstract

Obesity leads to ovarian dysfunction and the establishment of local leptin resistance. The aim of our study was to characterize the levels of NOD-like receptor protein 3 (NLRP3) inflammasome activation in ovaries and liver of mice during obesity progression. Furthermore, we tested the putative role of leptin on NLRP3 regulation in those organs. C57BL/6J female mice were treated with equine chorionic gonadotropin (eCG) or human chorionic gonadotropin (hCG) for estrous cycle synchronization and ovary collection. In diet-induced obesity (DIO) protocol, mice were fed chow diet (CD) or high-fat diet (HFD) for 4 or 16 weeks, whereas in the hyperleptinemic model (LEPT), mice were injected with leptin for 16 days (16 L) or saline (16 C). Finally, the genetic obese leptin-deficient *ob/ob* (+/? and −/−) mice were fed CD for 4 week. Either ovaries and liver were collected, as well as cumulus cells (CCs) after superovulation from DIO and LEPT. The estrus cycle synchronization protocol showed increased protein levels of NLRP3 and interleukin (IL)-18 in diestrus, with this stage used for further sample collections. In DIO, protein expression of NLRP3 inflammasome components was increased in 4 week HFD, but decreased in 16 week HFD. Moreover, NLRP3 and IL-1β were upregulated in 16 L and downregulated in *ob/ob.* Transcriptome analysis of CC showed common genes between LEPT and 4 week HFD modulating NLRP3 inflammasome. Liver analysis showed NLRP3 protein upregulation after 16 week HFD in DIO, but also its downregulation in *ob/ob−/−*. We showed the link between leptin signaling and NLRP3 inflammasome activation in the ovary throughout obesity progression in mice, elucidating the molecular mechanisms underpinning ovarian failure in maternal obesity.

## Introduction

Obesity leads to chronic systemic inflammation, a process mostly promoted by the continuous expansion of adipose tissue (Hotamisligil and Erbay, 2008; Odegaard and Chawla, 2008). Importantly, obesity is strongly linked to reproductive failure and infertility in women (Chu et al., 2007). The ovaries of mice fed a high-fat diet (HFD) show increased apoptosis and fewer mature oocytes (Jungheim et al., 2010). Furthermore, we have recently identified a striking link between maternal body weight in diet-induced obese (DIO) mice and global gene expression in cumulus cells (Wołodko et al., 2020). Other readouts of ovarian failure during maternal obesity comprise lipotoxicity, endoplasmic reticulum (ER) stress, and mitochondrial dysfunction (Wu et al., 2010). Ultimately, impaired ovarian function in obese mothers determines poor oocyte quality and abnormal embryo development (Minge et al., 2008), as obesity in women has been largely associated with failure in embryo implantation and abortion (Chu et al., 2007; Robker, 2008; Penzias, 2012). Thus, ovarian failure and decreased oocyte quality contribute to infertility in obese mothers.

Maternal obesity has other implications beyond infertility. The impact of maternal of obesity in offspring health represents a greater economic and social burden. Fetal congenital abnormalities were reported in obese mothers (Samuelsson et al., 2008). Furthermore, reports suggested also increased predisposition to cardiovascular disease (Loche et al., 2018) and type 2 diabetes (Blüher, 2019) in the offspring born from obesity mothers. Therefore, maternal obesity presents major long-term implications for offspring development and health.

The white adipose tissue (WAT) is a major endocrine organ and, alongside skeletal muscle and the liver, regulates whole body insulin sensitivity and glucose homeostasis (Czech, 2020). Maternal obesity is associated with systemic hormonal imbalance, largely characterized by excessive expansion of white adipose tissue and increased circulating levels of insulin, cholesterol, and leptin (Wołodko et al., 2020), among others. We have recently demonstrated that the establishment of leptin resistance in the ovaries of mice treated with HFD (Wołodko et al., 2020) was mostly mediated by suppressor of cytokine signaling 3 (SOCS3). Hence, early increase in ovarian leptin signaling and overexpression of SOCS3 protein after 4 weeks of DIO, was followed by failure in leptin receptor (ObR) b activation and leptin resistance after 16 week DIO (Wołodko et al., 2020). Furthermore, leptin is a well-known regulator of ovarian function (Karlsson et al., 1997; Ryan et al., 2002), with the aforementioned changes in leptin signaling in the ovaries of obese mothers potentially affecting major functional events like follicular pool activation, oocyte maturation, and ovulation (Wołodko et al., 2021).

The inflammasome is a large intracellular protein complex that contains a cytosolic pattern recognition receptor. Among NOD-like receptors (NLR), the NLR protein 3 (NLRP3) inflammasome has been best characterized as a complex of proteins responsible for controlling the activity of two proinflammatory cytokines interleukin (IL)-1β and IL-18 (Martinon et al., 2002; Davis et al., 2011; De Nardo and Latz, 2011). Activation of the pattern recognition receptor NLRP3 can be accomplished through two major signals: (i) priming signal, induced by the Toll-like receptor (TLR)/nuclear factor (NF)-κB pathway, and (ii) pathogen-associated molecular patterns (PAMPs) and damage-associated molecular patterns (DAMPs) leading to assembly of inflammasome (Martinon et al., 2002; Lamkanfi and Dixit, 2014). Both mechanisms lead to the recruitment of the adapter apoptosis-associated speck-like protein containing a C-terminal caspase recruitment domain (ASC), resulting in the activation of pro-caspase-1 (CASP1) and cleavage into the active form (Davis et al., 2011). The formation and activation of the inflammasome is possible through ASC, which links NLRP3 to CASP1 by means of its pyrin and caspase recruitment domain motifs (Martinon et al., 2006). Finally, activated CASP1 is known to process the maturation of IL-1β and IL-18 into active cytokines (Lamkanfi, 2011). Importantly, obesity and insulin resistance (IR) have been associated with inflammation and subsequent activation of NLRP3 inflammasome (Traba and Sack, 2017). The onset of inflammasome activation was also shown to be mediated by factors like glucose, ceramide, uric acid, or lipopolysaccharide (LPS) (Stienstra et al., 2012; Traba and Sack, 2017). Furthermore, secondary signals like extracellular ATP inducing K+ efflux, DAMPs/PAMPS leading to reactive oxygen species (ROS) production can also activate NLRP3 inflammasome (Shao et al., 2015; Tõzsér and Benkõ, 2016). Saturated free fatty acids (FFAs) were equally linked to inflammasome activation through both signals (Wen et al., 2011), as increased levels of FFAs are a general feature of obesity, IR, or type-2 diabetes (Boden, 2002; Krebs and Roden, 2005). More recently, a link has been also established between NLRP3 inflammasome activation and levels of leptin signaling in various cellular contexts (Fu et al., 2017), substantiating leptin proinflammatory role (Cauble et al., 2018).

A recent report has shown the presence of NLRP3 inflammasome components at ovarian level during follicular development in mice, suggesting its involvement in ovulation (Z. Zhang et al., 2019). Most importantly, NLRP3 was also suggested to be involved in the pathophysiology of polycystic ovary syndrome (PCOS) (Rostamtabar et al., 2020). Therefore, we presently hypothesize that the regulation of NLRP3 in the ovary of obese mice is mediated by local leptin signaling. We first confirmed that NLRP3 inflammasome expression profile changed in the ovaries of cyclic mice. Subsequently, we revealed that NLRP3 inflammasome components were differently expressed in the ovaries of 4- and 16- week DIO mice. Furthermore, using a mouse model of pharmacological hyperleptinemia (LEPT) and a genetic obese mouse, which lacks leptin (*ob/ob*), we demonstrated the association between levels of leptin signaling and NLRP3 inflammasome activation in the ovary of obese mice. Moreover, we re-analyzed the transcriptome of cumulus cells (CCs) from DIO and LEPT models and concluded that leptin treatment upregulated genes associated with NLRP3 inflammasome in CCs. Finally, we studied the NLRP3 inflammasome component expression in the liver of DIO, LEPT, and *ob/ob* mice. We observed a consistent downregulation in NLRP3 inflammasome activity in *ob/ob*, which are obese and lack leptin, denoting once more the important link between NLRP3 and leptin activity.

## Materials and Methods

### Animals and Protocols

Breeding pairs were purchased from Jackson Laboratories (Bar Harbor, ME, United States). Female C57BL/6J (B6) mice (8-week old) and B6.Cg-Lepob/J (*ob/ob*) were housed in the Animal Facility of Institute of Animal Reproduction and Food Research, Polish Academy of Sciences in Olsztyn. Mice were housed with free access to food and water for the duration of the study (humidity 50 ± 10%; 23°C; 12L:12D cycle). All procedures were approved by the Local Animal Care and Use Committee for the University of Warmia and Mazury in Olsztyn. Guidelines for animal experiments followed EU Directive 2010/63/EU. Throughout the experiments, mice were monitored for any sings of welfare or disease. At 8 week of age, mice were subjected to various protocols.

For hormonal treatment protocol, the estrous cycle was monitored after vaginal cytology analysis. Cells were collected on a glass slide and stained with Diff Quik^®^ kit (Medion Diagnostics AG, Switzerland, DQ-ST). Estrus (E) was characterized by cornified epithelium cells, metestrus by both cornified cells and leukocytes, diestrus (D) by predominant leukocytes, and proestrus by nucleated cells, as previously described (Kyrönlahti et al., 2011). Subsequently, mice received either eCG or hCG, as previously described (Hasegawa et al., 2016). One group of female B6 mice (seven to eight mice/group) was injected in E with equine chorionic gonadotropin (eCG, G4877; 5 IU, Sigma Aldrich) followed by human chorionic gonadotropin (hCG, Chorulon, 5 IU, MSD Animal Health) 48 h after, and tissues were collected 18–20 h later in E. In the second group (seven to eight mice/group), animals were injected with hCG, and tissues were collected 16–18 h later in D. In the DIO model, mice (8–10/group) were placed on standard chow diet (CD, #5053, Picolab Rodent diet 20 with 13% of calories) or HFD (59% of calories, AIN-76A with 33% hydrogenated coconut oil; LabDiet) for 4 or 16 week. In the hyperleptinemic model (8–10 mice/group), high circulating levels of leptin were obtained through intraperitoneal administrations of leptin twice (100 μg/day injected at 09:00 and 21:00), while the control group received saline (Recombinant Mouse Leptin, GFM26, Cell Guidance Systems). Regarding the *ob/ob* model, mice (8–10 mice/group) were kept on CD until 12 week of age.

### Immunohistochemistry and Immunofluorescence

Both immunohistochemistry (IHC) and immunofluorescence (IF) followed the protocols previously described (Wołodko et al., 2020). Briefly, mouse ovaries were collected during the D stage, fixed in 4% paraformaldehyde, and stored at 4°C. On the following day, the tissues were dehydrated in ethanol. Paraffin embedded tissues were then cut in 5-μm sections and mounted on a glass slide. After deparaffinization in xylene, samples were rehydrated in increasing ethanol concentrations. For antigen retrieval, sections were boiled in citrate buffer (10 mM, pH = 6). Next, sections were incubated with BSA (A2153; Sigma Aldrich) for 1 h at room temperature (RT), followed by incubation with rabbit polyclonal antibody against NLRP3 (1:200, ab214185; Abcam) overnight at 4°C. In order to test the specificity of the primary antibody, sections were also incubated with rabbit polyclonal anti-immunoglobulin G (IgG, ab37415; Abcam) or without primary antibody (negative controls). On the following day, sections were washed and incubated for 1 h at RT with peroxidase-conjugated goat anti-rabbit IgG polyclonal antibody (1:100, 410972; Dako). Finally, slides were incubated in 3,3-diaminobenzidine (DAB) for 15 s before visualization. The sections were then counterstained for 2 min using hematoxylin (MHS16; Sigma Aldrich). Stained sections were dehydrated and mounted under glass coverslips.

Concerning the immunofluorescence protocol, after deparaffinization in xylene, samples were in increasing ethanol concentrations. Sections were then permeabilized in 0.3% Triton X-100 (T8787; Sigma Aldrich) two times for 5 min at RT. Antigen retrieval was done after heating the sections in citrate buffer (10 mM, pH = 6). Blocking was done with BSA (A2153; Sigma Aldrich) with 0.3 M glycine (G8898; Sigma Aldrich) in phosphatase-buffered saline (PBS)–0.1% Tween 20 (P7949; Sigma Aldrich) (PBST) solution for 2 h, at RT. In order to minimize the autofluorescence, tissues were treated with 0.3% Sudan Black (199664; Sigma Aldrich) (in 70% ethanol) for 10 min at RT. Subsequently, sections were washed eight times for 5 min in PBST (0.1% Tween20). The sections were then incubated with rabbit polyclonal antibody against NLRP3 (1:100, NBP2-12446; Novus Biologicals) overnight at 4°C. The negative controls were after incubating the slides with rabbit polyclonal anti-immunoglobulin G (IgG, ab37415; Abcam) or without primary antibody (negative controls). On the following day, the sections were washed six times for 5 min in PBST at RT, followed by incubation with the secondary antibody cyanine 3 (Cy3)-AffiniPure donkey polyclonal anti-rabbit IgG (H+L) (1:400, 711-165-152; Jackson ImmunoResearch) for 2 h at RT. The sections were washed six times for 5 min in PBST at RT. In order to visualize the nucleus, DAPI (dilution 1:100 with PBS, D9542-10MG; Sigma Aldrich) was added for 30 min at RT. Finally, the slides were covered with Vectashield medium (H-1000; Vector Laboratories) and stored at −20°C. Images were captured using Zeiss Axio Observer System (Carl Zeiss, Germany) with × 20/0.4 NA, and confocal microscope (Zeiss LSM 800) with × 40/1.2 or × 63/1.4 NA oil immersion objectives. For examination of the slides, an Axio Observer Systems Z1 microscope (Carl Zeiss Microscopy GmbH, Germany) and the Zeiss ZEN 2.5 lite Microscope Software (Carl Zeiss, Germany) were used.

### Protein Extraction and Western Blotting Analysis

Protein expression in mouse ovary and liver was assessed by Western blotting. Ovaries and livers were homogenized with RIPA buffer (R0278; Sigma Aldrich) after adding protease inhibitors (phenylmethylsulfonyl fluoride, PMSF and Protease Inhibitor Cocktail, P8340; Sigma-Aldrich) and phosphatase inhibitors (Pierce Phosphatase Inhibitor Mini Tablets 88667; Thermo Fisher Scientific) and incubating on ice for 1 h. After centrifugation (20,000 × *g*, 15 min, 4°C), the supernatants were collected, and protein concentration was determined with the Smith et al. (1985) copper/bicinchoninic assay [Copper (II) Sulfate, C2284; Sigma and Bicinchoninic Acid Solution, B9643; Sigma Aldrich]. Samples were run (40 μg of protein) on 10–18% polyacrylamide gels. After transfer, the membranes were blocked in PBS solution containing 3% powdered milk for 1 h. Immunoblotting was performed using the primary antibodies NLRP3 (AG-20B-0014-C100; Adipogen), CASP1 (ab108362; Abcam), IL-18 (ab71495; Abcam), β-actin (A2228; Sigma Aldrich) and glyceraldehyde 3-phosphate dehydrogenase (GAPDH, ab9485; Abcam) on nitrocellulose (10600009; GE Healthcare Life Science) or polyvinylidene fluoride (PVDF) membrane (IPVH00010; Merck Millipore). Primary antibodies were incubated overnight at 4°C. The following day, proteins were detected by incubating the membranes with polyclonal anti-mouse horseradish peroxidase (HRP)-conjugated secondary (1:10,000, 31430; Thermo Fisher Scientific), polyclonal anti-rabbit HRP-conjugated secondary (1:20,000, 31460; Thermo Fisher Scientific), polyclonal anti-mouse alkaline phosphatase-conjugated secondary (1:10,000, 31321; Thermo Fisher Scientific), and polyclonal anti-rabbit alkaline phosphatase-conjugated secondary (1:10,000, A3687; Sigma Aldrich) antibodies, for 1.5 h in the chemiluminescence method or 2.5 h in the colorimetric method at RT. All antibody specifications are summarized in Table 1. Immunocomplexes were visualized subsequently using chemiluminescence detection reagent (SuperSignal West Femto kit, 34095; Thermo Fisher Scientific) or chromogenic substrate NBT/BCIP diluted 1:50 (11681451001; Roche) in alkaline phosphate buffer. Band density for each of the target protein was normalized against β-actin for NLRP3 and IL-18, while GAPDH was used for CASP1 as a reference protein. Finally, bands were quantified using the ChemiDoc or VersaDoc MP 4000 imaging system (Bio-Rad). Quantitative measurements of blot intensity were performed using ImageLab software.

**TABLE 1:** Specification of antibodies used for Western blotting.

**Antibody name and specificity**	**Company, Cat No., RRID No.**	**Antibody dilution**
Mouse monoclonal against NLR family pyrin domain-containing 3 (NLRP3)	AdipoGen Cat# AG-20B-0014, RRID:AB_2490202	1:1,000
Rabbit monoclonal against caspase 1 (CASP1)	Abcam Cat# ab108362, RRID:AB_10858984	1:1,000
Rabbit polyclonal against interleukin-18 (IL-18)	Abcam Cat# ab71495, RRID:AB_1209302	1:250
Mouse monoclonal against β-actin	Sigma-Aldrich Cat# A2228, RRID:AB_476697	1:10,000
Rabbit polyclonal against glyceraldehyde 3-phosphate dehydrogenase (GAPDH)	Abcam Cat# ab9485, RRID:AB_307275	1:2,500
Goat anti-mouse IgG (H+L) secondary antibody, HRP	Thermo Fisher Scientific Cat# 31430, RRID:AB_228307	1:1,000
Goat anti-rabbit IgG (H+L) secondary antibody, HRP	Thermo Fisher Scientific Cat# 31460, RRID:AB_228341	1:20,000
Goat anti-mouse IgG (H+L) secondary antibody, AP	Thermo Fisher Scientific Cat# 31321, RRID:AB_10959407	1:1.000

### Total RNA Isolation and cDNA Synthesis

Total RNA was extracted from whole ovary and 10 mg of liver, using TRI reagent (T9424; Sigma Aldrich) following the instructions of the manufacturer. RNA samples were stored at −80°C. Concentration and quality of RNA was determined spectrophotometrically, and the ratio of absorbance at 260 and 280 (A_260/280_) was analyzed confirming good RNA quality. Subsequently, 2 μg of RNA was reverse transcribed into cDNA using Maxima First Strand cDNA Synthesis Kit for RT-qPCR (K1642; Thermo Fisher Scientific) (Galvão et al., 2012).

### Real-Time PCR

Real-time PCR assays were performed in a 7900 Real-time System (Applied Biosystems), using a default thermocycler program for all genes: a 10-min preincubation at 95°C was followed by 45 cycles of 15 s at 95°C and 1 min at 60°C. A further dissociation step (15 s at 95°C, 15 s at 60°C, and 15 s at 95°C) ensured the presence of a single product. *Ribosomal protein L37 (Rpl37)* was chosen as a housekeeping gene and quantified in each real-time assay together with the target gene. Based on gene sequences in GenBank (National Center for Biotechnology Information), the primers for *Nlrp3*, *Casp1*, *Il-1*β, *Il-18*, *Asc*, *Il-10*, and *Tnf*, which sequences are presented in Table 2, were designed using Primer Express 3.0 software (Applied Biosystems). All reactions were carried out in duplicates in 384-well plate (4309849; Applied Biosystems) in 12 μl of total solution volume (Galvão et al., 2014). The data were analyzed using the real-time PCR Miner algorithm (Zhao and Fernald, 2005).

**TABLE 2:** Specific primers used for quantitative real-time PCR.

**Gene name**	**Gene symbol**	**GenBank accession No.**	**Sequences 5′–3′**	**Length (base pairs)**
NLR family pyrin domain-containing 3	*Nlrp3*	NM_145827.4	F: TGGATGGGTTTGCTGGGATA R: TGCTTGGATGCTCCTTGACC	190
Caspase 1	*Casp1*	NM_009807.2	F: CATGCCGTGGAGAGAAACAA R: GGTGTTGAAGAGCAGAAAGCAA	151
Interleukin-1β	*IL-1*β	NM_008361.4	F: TTGACGGACCCCAAAAGATG R: GCTTCTCCACAGCCACAATGA	144
Interleukin-18	*Il-18*	NM_008360.2	F: GAAGAAAATGGAGACCTGGAATCA R: TCTGGGGTTCACTGGCACTT	157
Apoptosis-associated speck-like protein-containing A CARD	*Asc*	NM_023258.4	F: GCTTAGAGACATGGGCTTACAGGA R: CCAGCACTCCGTCCACTTCT	179
Interleukin-10	*Il-10*	NM_010548.2	F: CCTGGGTGAGAAGCTGAAGAC R: CTGCTCCACTGCCTTGCTCT	91
Tumor necrosis factor	*Tnf*	NM_001278601.1	F: GCCACCACGCTCTTCTGTCT R: TGAGGGTCTGGGCCATAGAA	106
Ribosomal protein L37	*Rpl37*	NM_026069.3	F: CTGGTCGGATGAGGCACCTA R: AAGAACTGGATGCTGCGACA	108

### Enzyme-Linked Immunosorbent Assay Immunoassay

The concentrations of IL-1β in tissue extracts of ovaries and livers were determined using an IL-1 beta Pro-form Mouse Uncoated ELISA kit (88-8014-22; Thermo Fisher Scientific) following the instructions of the manufacturer. The standard curve concentrations ranged from 25 to 3,000 ng/m, and interassay coefficient variation (CV) was 7.27%.

### RNA-Seq Data From Cumulus Cells

We used RNA-seq data from CCs previously produced by Wołodko et al. (2020). The dataset is publicly available under the GEO accession number GSE180300^1^. Library generation was previously described (Wołodko et al., 2020). Briefly, we collected approximately 50 CCs per animal, after superovulation, and RNA-seq libraries were generated using a Smart-seq2 oligo-dT method. Read counts were normalized by size factor and log-2 transformed. Partial correlations were used to determine coexpressing genes (cor > 0.9) using the corpcor package (Schäfer and Strimmer, 2005). Network statistics were calculated using the igraph package (Csárdi and Nepusz, 2006). Network visualization was done with Cytoscape (Shannon et al., 2003).

### Statistical Analysis and Data Presentation

Statistical analyses were performed using the GraphPad Prism Software (Version 9.01, GraphPad Software, Inc.; La Jolla, CA, United States). Sample normal distribution was determined using the D’Agostino–Pearson omnibus test. Mann–Whitney test, simple *t*-test, or multiple unpaired *t*-test were used to analyze the data, and statistical significance was calculated with Bonferroni–Sidak corrections for multiple comparison, depending on the experiment (details in figure legend). Results were presented as means with standard deviation. Differences between means for all tests were considered statistically significant if *p* < 0.05.

## Results

### NOD-Like Receptor Protein 3 Inflammasome Components Expression Change in the Ovary of Cyclic Mice

**FIGURE 1:**
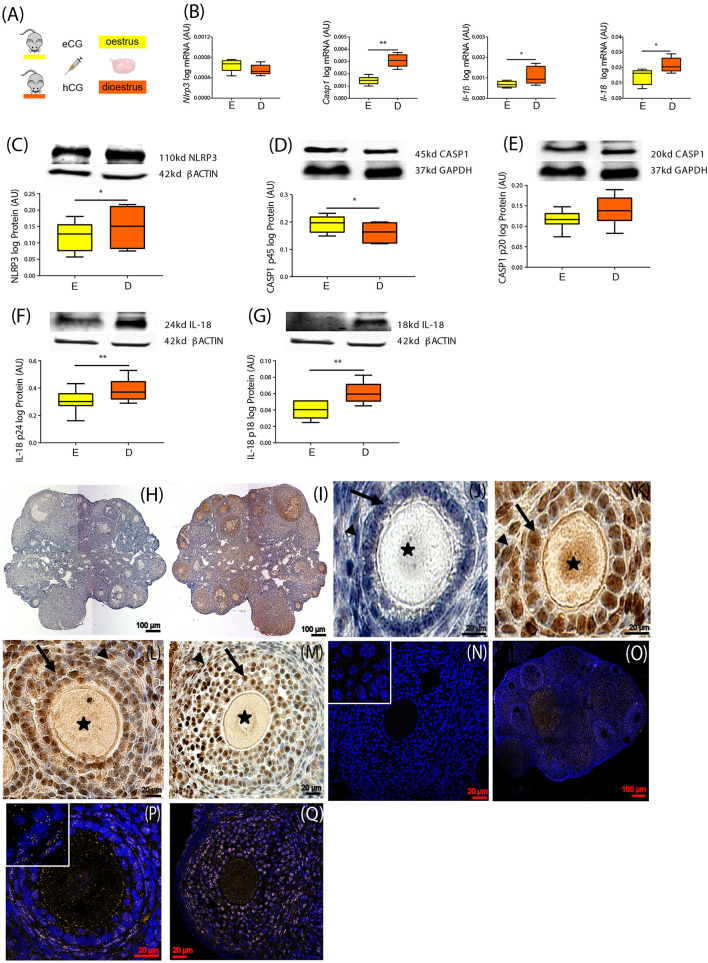
Characterization of NOD-like receptor protein 3 (NLRP3) expression in the ovary of cyclic mice. **(A)** Experimental design: estrous cycle synchronization with equine chorionic gonadotropin (eCG) and human chorionic gonadotropin (hCG) as previously described (Hasegawa et al., 2016). Ovaries were collected from animals in the estrus **(E)** or diestrus **(D)** stage of the cycle. Quantification of mRNA levels of **(B)** *Nlrp3*, caspase-1 (*Casp1*), interleukin-1β (*Il-1*β), and interleukin-18 (*Il-18*) by real-time PCR. Abundance of **(C)** NLRP3, **(D)** pro CASP1 p45, **(E)** CASP1 p20, **(F)** pro IL-18 p24, and **(G)** IL-18 p18 protein during **(E,D)** measured by Western blotting analysis. Data were normalized to ribosomal protein L37 (*Rpl37*) mRNA expression and β-actin of or glyceraldehyde 3-phosphate dehydrogenase (GAPDH) protein expression. Bars represent mean ± SEM. Statistical analysis between groups was carried out using Mann–Whitney. Number of samples: *n* = 6–7 for real-time PCR analysis and *n* = 7–8 immunoblots. Asterisks indicate significant differences (^∗^*p* < 0.05; ^∗∗^*p* < 0.01). Representative immunohistochemistry (IHC) staining (*n* = 2–3) of NLRP3 protein during follicular development in the mouse ovary. Positive staining in brown, counterstaining with hematoxylin. **(H,J)** Negative control incubated with secondary antibody. Localization of NLRP3 in **(I)** whole ovary of 16 weeks (wk) mice fed chow diet (CD), **(K)** primary follicles of 16- week CD mice, **(L)** secondary follicles of 16- week high-fat diet (HFD) mice, and **(M)** preantral follicles of 16- week CD mice. Staining was detected in granulosa (GC) and theca cells (TC). Faint staining was observed in the oocytes of all stages of folliculogenesis. Arrows denote GC, arrowheads denote TC, and asterisks denote oocytes. The IHC staining was confirmed by immunofluorescent localization of NLRP3. Positive staining in orange, nuclear counterstaining with DAPI in blue. **(N)** Negative control 16- week CD stained with polyclonal rabbit IgG. NLRP3 localized in **(O)** whole ovary, **(P)** secondary follicles of 16- week CD mice, **(Q)** preantral follicles of 16- week HFD mice. Inserts on the top left corners represent magnifications of GC. Scale bars represent 20 or 100 μm. AU, arbitrary units.

We first sought to characterize the expression of NLRP3-induced inflammasome components in the ovaries of mice throughout the estrous cycle. Fifteen female 8-week-old C57BL/6 (B6) mice were treated with hormones in order to synchronize the estrous cycle (Figure 1A). Ovaries were collected in the E and D stage and further processed for mRNA or protein expression analysis, respectively. Real-time PCR analysis (*n* = 6–7/group) revealed increased levels of *Casp1*, *Il-1*β, and *Il-18* mRNA in the D stage (Figure 1B, *p* < 0.05). Moreover, Western blotting (*n* = 7–8/group) revealed increased NLRP3 protein expression in D (Figure 1C, *p* < 0.05), as well as the pro-peptide (p24) and mature form (p18) of IL-18 (Figures 1F,G; *p* < 0.01). Regarding CASP1, the long form (p45) was decreased in D (Figure 1D; *p* < 0.05), but no significant changes were observed for the active CASP1 (p20) (Figure 1E). These results suggest the activation of NLRP3 inflammasome in the D stage, through upregulation of NLRP3 and its downstream mediator IL-18. Next, we characterized the cellular distribution of NLRP3 protein in the ovaries collected from mice in D, using IHC and IF (*n* = 2–3/group). We confirmed that NLRP3 protein was visible in ovarian follicles, corpora lutea, and interstitial space (Figure 1I). On the other hand, a closer observation of IHC sections revealed staining in granulosa cells (GC) and theca cells (TC), as well as in oocytes, in all developmental follicular stages (Figures 1K–M). Negative controls stained exclusively with secondary antibodies did not reveal any brown staining (Figures 1H,J). The specificity of our IHC staining was corroborated by IF, in which a clear yellow staining was observed in GC, TC, and oocytes (Figures 1O–Q). Negative control stained with rabbit immunoglobulin type G (IgG) confirmed no staining (Figure 1N). Our results not only confirmed the presence of NLRP3 protein in GC, TC, and oocytes by IHC and IF, but also confirmed the upregulation of NLRP3 in the ovaries of eCG-treated mice. Therefore, in subsequent experiments, collections were consistently performed in D.

### Activation of NOD-Like Receptor Protein 3-Induced Inflammasome in the Ovary of Diet-Induced Obesity Mice

**FIGURE 2:**
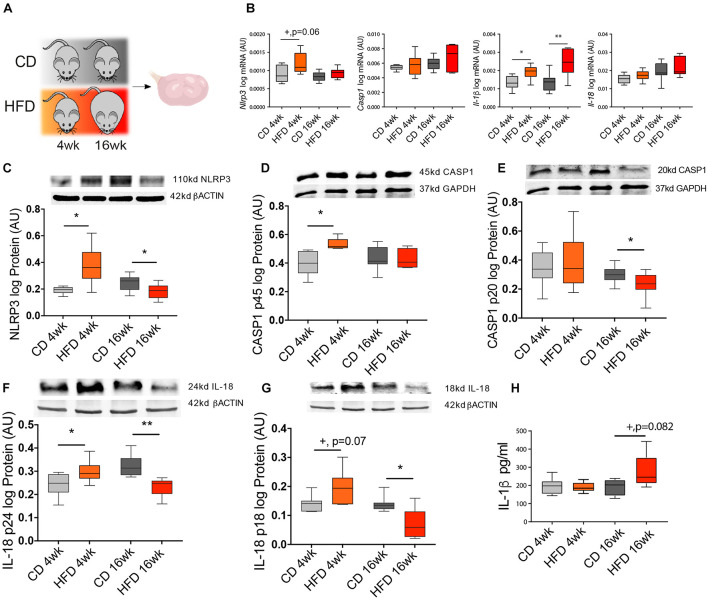
Diet-induced obesity (DIO) changes NLRP3 expression in the ovary. **(A)** Experimental design: Mice were fed either chow diet (CD) or high-fat diet (HFD) for 4 or 16 weeks (wk) and ovaries were collected during the diestrus stage. Quantification of **(B)** *Nlrp3*, *Casp1*, *Il-1*β, and *Il-18* mRNA by real-time PCR. Abundance of **(C)** NLRP3, **(D)** pro CASP1 p45, **(E)** CASP1 p20, **(F)** pro IL-18 p24, **(G)** IL-18 p18 protein measured by Western blotting and **(H)** IL-1β protein measured by enzyme-linked immunosorbent assay (ELISA) in ovarian extracts collected from DIO mice. mRNA level was normalized with ribosomal protein L37 (*Rpl37*) value and protein expression with β-actin or glyceraldehyde 3-phosphate dehydrogenase (GAPDH) level. Bars represent mean ± SEM. Differences between control and treatment groups analyzed with Mann–Whitney in real-time PCR and ELISA and multiple *t*-test for Western blotting. Number of samples: *n* = 6–8 for real-time PCR, *n* = 7–8 for immunoblots, and *n* = 4–6 for ELISA. Asterisks indicate significant differences (*p < 0.05; **p < 0.01; +*p* = 0.06; +*p* = 0.07 or +*p* = 0.082—all values indicated). AU, arbitrary units.

In the following experiment, we tested the effects of short- (4 week) vs. long-term (16 week) HFD treatment on NLRP3-induced inflammasome activation in the ovary of mice (Figure 2A). Throughout the mouse DIO protocol, the average body weight (BW) was recorded every 4 week (Table 3). After collection, ovaries were processed for mRNA and protein expression analysis. Real-time PCR analysis (*n* = 6–8/group) revealed increased mRNA of *Nlrp3* after 4-week HFD (Figure 2B, *p* = 0.06), whereas *Il-1*β levels were increased after both 4 and 16 week of HFD (Figure 2B, *p* < 0.05). Regarding Western blotting analysis (*n* = 7–8/group), we found that the expression of NLRP3, CASP1 p45, and pro IL-18 p24 were increased in the 4-week HFD group, compared with the control group (Figures 2C,D,F, *p* < 0.05, respectively). The opposite pattern was seen after 16-week HFD, with the downregulation of NLRP3 expression, the mature form of CASP1 p20 and both forms of IL-18 (p24 and p18) (Figures 2C,E–G, *p* < 0.05). Finally, we also confirmed that IL-1β protein level was upregulated in 16-week HFD by enzyme-linked immunosorbent assay (ELISA) (*n* = 4–6) (Figure 2H, *p* = 0.082). We presently showed that despite the upregulation of NLRP3, the pro-proteins IL-18 (p18, p24) and CASP1 (p45) after 4-week HFD treatment, the expression profile of NLRP3, CASP1 (p20), and both forms of IL-18 (p18, p24) were consistently downregulated after a 16-week HFD treatment.

**TABLE 3:** Body weight measurement of three mouse models.

	**0 week**	**4 week**	**8 week**	**12 week**	**16 week**
**CD**	17.0 (± 0.6) g	19.7 (± 1.0) g	20.0 (± 0.9) g	20.9 (± 1.0) g	22.6 (± 2.5) g
**HFD**	19.4 (± 0.8) g	24.8 (± 2.5) g****	29.1 (± 3.6) g****	33.1 (± 4.3) g****	37.6 (± 3.7) g****

	**0 day**	**3 days**	**9 days**	**12 days**	**16 days**

**C**	21.7 (± 1.7) g	22.8 (± 2.0) g	22.1 (± 2.1) g	21.5 (± 1.6) g	21.9 (± 1.9) g
**L**	21.8 (± 1.9) g	20.5 (± 1.9) g	19.0 (± 1.4) g**	18.5 (± 1.2) g***	19.4 (± 1.7) g

	**8** week	**9 week**	**10 week**	**11 week**	**12 week**

**ob/ob +/?**	20.9 (± 1.6) g	21.5 (± 1.6) g	22.2 (± 1.6) g	22.8 (± 2.1) g	22.7 (± 2.1) g
**ob/ob –/–**	39.8 (± 5.4) g****	43.1 (± 5.0) g****	45.0 (± 4.7) g****	47.4 (± 5.7)****	48.8 (± 4.0) g****

DIO, Diet-induced obese; CD, mice were fed chow diet; HFD, high-fat diet (ii) pharmacologically hyperleptinemic mice were treated with saline (C) or leptin (L) for 16 days; (iii) genetically obese mice lacking leptin (ob/ob −/−) and control group (ob/ob +/?). Values presented in grams (g) of body weight and measurements made after weeks (wk) or days (d) within the specific protocol. Statistical analysis between groups was carried out using simple t-test. Asterisks indicate significant differences (**p < 0.01; ***p < 0.001; ****p < 0.0001).

### Leptin Signaling in the Ovary Drives Activation of NOD-Like Receptor Protein 3 Inflammasome During Obesity Progression

**FIGURE 3:**
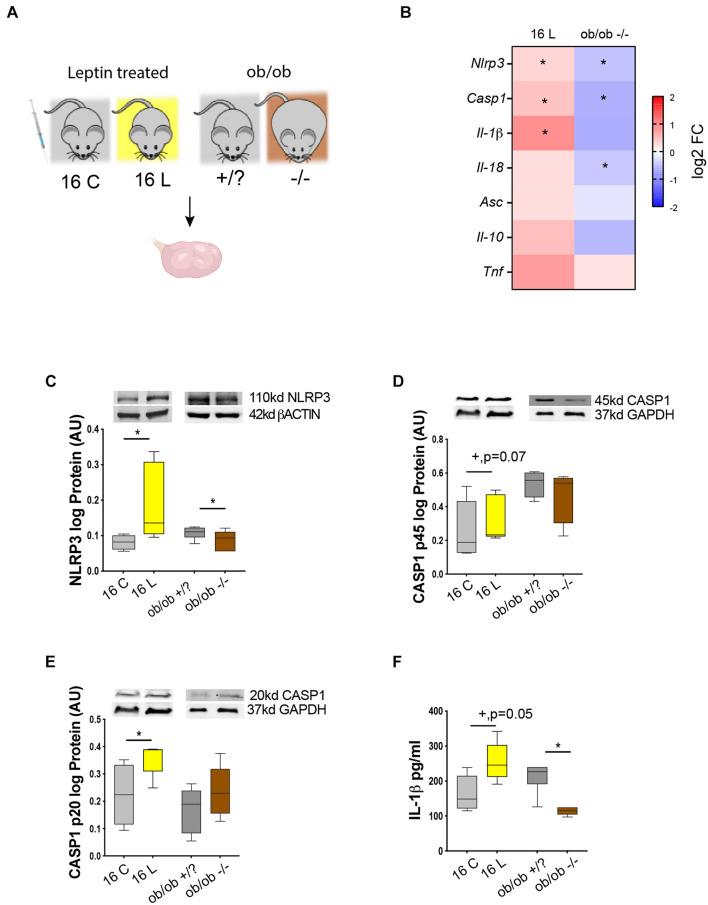
Leptin signaling in the ovary drive changes in NLRP3 during obesity. **(A)** Experimental design: (i) pharmacological hyperleptinemic model, mice were either injected with saline or 100 μg of leptin (L) for 16 days; (ii) genetic obesity model, mice lacking leptin (*ob/ob* −/−) or control group (*ob/ob* +/?). **(B)** Heatmap illustrating fold of change in expression of mRNA of genes *Nlrp3*, *Casp1*, *Il-1*β, *Il-18*, apoptosis-associated speck-like protein-containing A CARD (*Asc*), *Il-10*, and tumor necrosis factor alpha (*Tnf*) in hyperleptinemia and *ob/ob* models determined by real-time PCR. The scale matches colors to log 2 fold change (log2 FC) values. Abundance of **(C)** NLRP3, **(D)** pro CASP1 p45, **(E)** CASP1 p20 measured by Western blotting, and **(F)** IL-1β quantified by ELISA, in the mouse ovarian extracts. Level mRNA normalized with ribosomal protein L37 (*Rpl37*) value and protein expression with β-actin or glyceraldehyde 3-phosphate dehydrogenase (GAPDH). Bars represent mean ± SEM. Statistical analysis between groups was carried out using the Mann–Whitney test. Number of samples: *n* = 6–8 for real-time PCR analysis, *n* = 6–8 for immunoblots and *n* = 4–5 for ELISA. Asterisks indicate significant differences (*p < 0.05; +*p* = 0.07). AU, arbitrary units.

After temporally characterizing the expression profile of NLRP3-induced inflammasome components in the ovary of DIO mice, we further interrogated whether the activation of NLRP3 inflammasome was regulated by leptin signaling. Therefore, we analyzed the levels of NLRP3 inflammasome components in the ovaries of a previously validated mouse model of pharmacological hyperleptinemia, which presented increased systemic levels of leptin and increased leptin signaling in the ovary without obesity (Wołodko et al., 2020), and a genetically obese mouse *ob/ob* characterized by extreme obesity and lack of circulating leptin. In the pharmacological hyperleptinemic model (*n* = 8–10/group), B6 female mice were treated with leptin intraperitoneally 16 days (16 L), whereas controls were administered saline (16 C) (Wołodko et al., 2020). Moreover, in the *ob/ob* model (*n* = 8–10/group), control females (+/?) and homozygous mutant females (−/−) were kept on CD for 4 week (Figure 3A). Body weights of the mice were registered periodically (Table 3). Ovaries from all groups were collected and processed for mRNA and protein expression analysis. Real-time PCR analysis (*n* = 6–8/group) revealed an increase in *Nlrp3* and *Casp1* in 16 L, but a decrease in *ob/ob* −/− mice (Figure 3B, *p* < 0.05). Furthermore, the mRNA of *Il-1*β was upregulated in 16 L (Figure 3B, *p* < 0.05). Finally, the mRNA of *Il-18* was significantly downregulated in the *ob/ob* −/− group (Figure 3B, *p* < 0.05). With regard to protein expression (*n* = 6–8/group), we found that the 16 L group presented increased levels of NLRP3 (Figure 3C, *p* < 0.05), whereas the opposite pattern was observed in *ob/ob* −/− mice, compared with control groups (Figure 3C, *p* < 0.05). Accordingly, both pro-peptides CASP1 (p45) and CASP1 (p20) showed increased levels in 16 L (Figures 3D,E; *p* = 0.07 and *p* < 0.05, respectively); nonetheless, no significant changes were found in the *ob/ob* model. Importantly, IL-1β protein levels measured by ELISA (*n* = 4–5/group) were increased in 16 L, but decreased in *ob/ob* −/− (Figure 3F, *p* < 0.05). In this experiment, we revealed the functional link between leptin signaling and NLRP3 inflammasome component regulation in the ovary, with leptin treatment inducing the activation of NLRP3 and CASP1 with subsequent secretion of IL-1β. Furthermore, we confirmed the downregulation of NLRP3 expression in the ovaries of *ob/ob* −/−.

### Leptin Promotes Changes of NOD-Like Receptor Protein 3 Inflammasome Components Gene Expression in Cumulus Cells During Early Onset of Obesity

**FIGURE 4:**
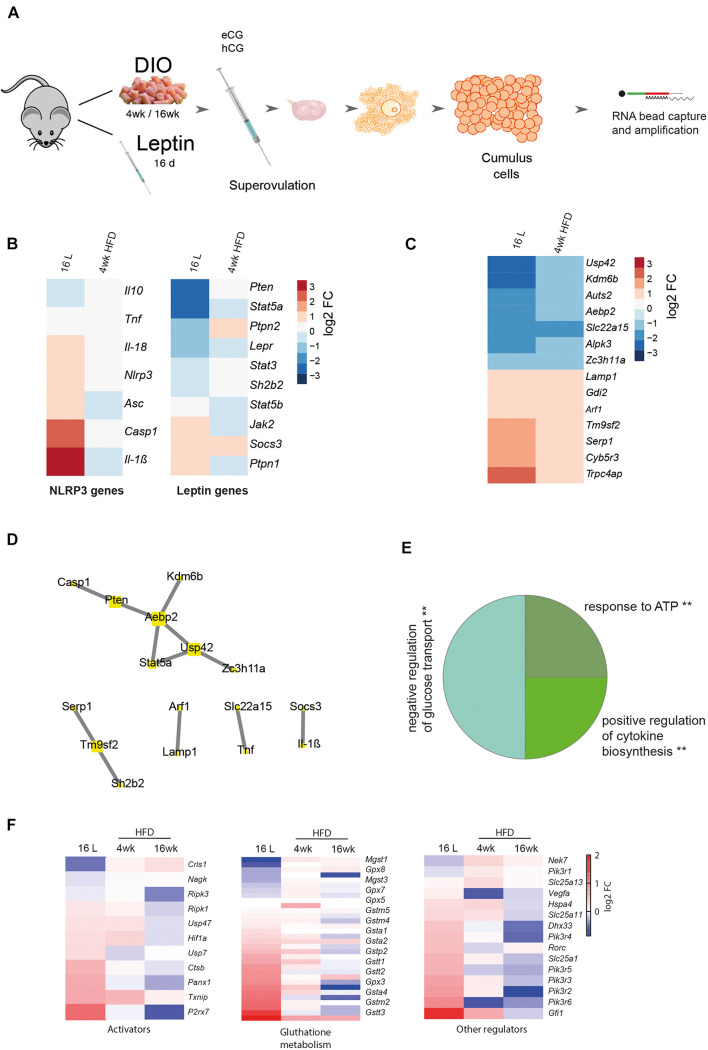
Cumulus cells transcriptome analysis from DIO and pharmacologically hyperleptinemic mice. **(A)** Experimental design: RNA-Seq analysis of differentially expressed genes in cumulus cells collected from mice: i) with DIO fed chow diet (CD) or high-fat diet (HFD) for 4 or 16 week (ii) with pharmacologically hyperleptinemic (LEPT) injected with saline **(C)** or 100 μg of leptin (L) for 16 days (16 L). **(B)** Heatmap illustrating expression of genes from leptin signaling pathway and NLRP3 inflammasome in 16 L and 4- week HFD normalized by control group fed CD or injected with saline, respectively. Downregulated genes in blue, upregulated genes in orange. The scale on the right matches colors to log 2 fold change (log2 FC) values. **(C)** Heatmap showing significant changes in major constitutive genes in both conditions 16 L and 4- week HFD. **(D)** Five main clusters in the network representing strong interaction between selected genes described in **(B)** and genes presented in **(C)**. **(E)** Pie chart that displays the main three gene ontology terms that were significantly enriched in cumulus cells in both conditions 16 L and 4- week HFD. Gene ontology analysis performed with Gene Ontology Enrichment Analysis and Visualization Tool. **(F)** Heatmaps showing conserved genes involved in NLRP3 inflammasome activation, glutathione metabolism, and other regulations. log2 FC of reads per million (RPM). Created with BioRender.com.

In this experiment, we examined whether the association between leptin signaling and NLRP3 inflammasome activation previously observed in whole ovaries holds true at the cellular level. We, therefore, analyzed particularly the somatic companions of the female gamete, the CCs. We started reanalyzing the RNA sequencing (RNA-seq) datasets from CCs from 4- and 16-week DIO and pharmacological hyperleptinemic model (Figure 4A; Wołodko et al., 2020). We confirmed the expression levels of leptin and NLRP3 pathway components for 16 L and 4-week HFD, and despite no changes in *Nlrp3* in CCs after 4-week HFD, the gene was upregulated in 16 L (Figure 4B). The low coverage of the samples (an average of 5.5 million reads) and the weak expression level of *Nlrp3* in CCs may account for the lack of changes in 4-week HFD. Nonetheless, the consistent upregulation of various components of the NLRP3 inflammasome, like *Nlrp3*, *Il-18*, *Casp1*, *Il-1*β, and *Asc* in 16 L is suggestive of the stimulatory effects of leptin on the expression level and activation of NLRP3 inflammasome genes also in CCs (Figure 4B). As previously shown, the DESeq analysis of 4-week DIO protocol revealed 997 differentially expressed genes (DEGs) in 4-week HFD, whereas for pharmacological hyperleptinemic model, a total of 2,026 DEGs were found in 16 L (Wołodko et al., 2020), in comparison with their control groups (*p* < 0.05; Wołodko et al., 2020). In the present analysis, we overlapped the DEGs from 4-week HFD and 16 L and identified seven genes either up- or downregulated in both conditions (Figure 4C). Subsequently, we integrated the 14 DEGs with the main components of NLRP3 and leptin signaling pathways (Wołodko et al., 2020), based on the correlation between their expression levels (*p* > 0.90), and obtaining five clusters (Figure 4D). Of note, one of the clusters revealed the gene interaction between *Casp1, phosphatase and tensin homolog (Pten)* and *signal transducer and activator of transcription 5a (Stat5a)*, while others showed a link between *Socs3* and *Il-1*β, known as an important axis involved in the mediation of immune response (Chaves de Souza et al., 2013). Importantly, other genes were highlighted in the network, as *solute carrier family 22 member 15 (Slc22a15)*, a cell membrane transporter and metabolic gene (Nigam, 2018), or *stress-associated endoplasmic reticulum protein 1 (Serp1)* involved in protein unfolding and stress response (Yamaguchi et al., 1999). Indeed, metabolic performance in the preovulatory follicle is tightly regulated and involves the crosstalk between GC and oocyte (Figure 4D; Wołodko et al., 2021). The gene ontology analysis for the presented network revealed three main events: negative regulation of glucose transport, positive regulation of cytokine biosynthesis, and response to ATP (Figure 4E, *p* < 0.05). Finally, we plotted a subset of genes known to directly activate the NLRP3 inflammasome signaling pathway (Weber et al., 2020), particularly regarding the regulation of glutathione, major mediator of NLRP3 signaling (Hughes et al., 2019), as well as other genes involved in the pathway regulation (Zhou et al., 2010; Barlan et al., 2011; Iyer et al., 2013; Mitoma et al., 2013; Wang et al., 2014; Kim et al., 2015; He et al., 2016b; Jo et al., 2016; Li et al., 2016; Wolf et al., 2016; Guglielmo et al., 2017; Hughes and O’Neill, 2018; Palazón-Riquelme et al., 2018; Shuvarikov et al., 2018; Billon et al., 2019; Hughes et al., 2019; Martine et al., 2019; Zhang et al., 2021). We confirmed a similar behavior between 16 L and 4-week HFD for those genes, in opposition to 16-week HFD (Figure 4F). Thus, systemic administration of leptin-activated genes from the NLRP3 inflammasome pathway in CCs, corroborating the functional link between leptin signaling and NLRP3 inflammasome activation in CCs of DIO mice.

### Activation of NOD-Like Receptor Protein 3-Induced Inflammasome in the Liver of Diet-Induced Obesity Mice

**FIGURE 5:**
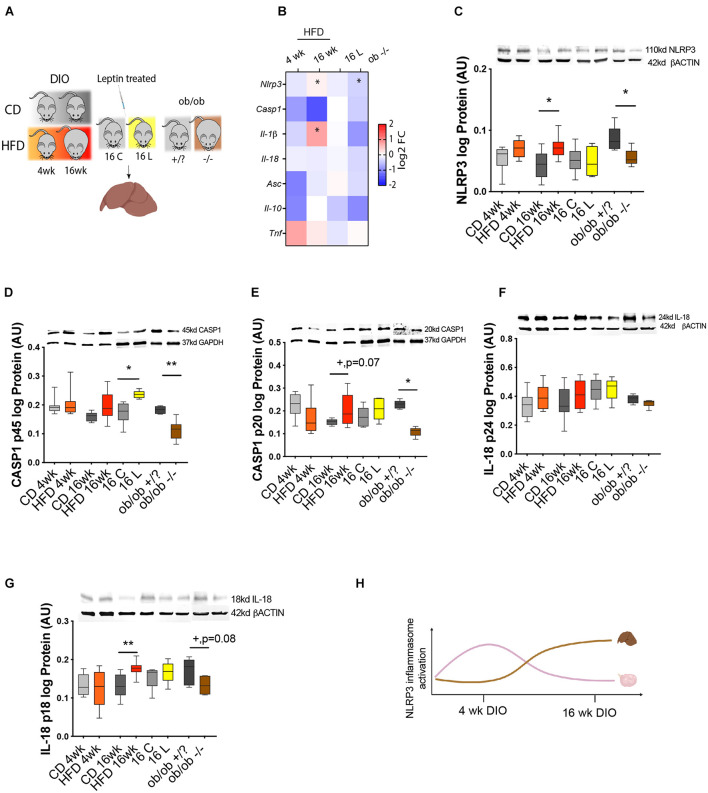
NLRP3 activity in the liver of DIO, hyperleptinemic, and genetically obese mice. **(A)** Experimental design: (i) DIO mice were fed CD or HFD for 4 or 16 week; (ii) pharmacologically hyperleptinemic mice were treated with saline **(C)** or 100 μg of leptin (L) for 16 days; (iii) genetically obese mice lacking leptin (*ob/ob*−/−) and control group (*ob/ob* +/?). **(B)** Heatmap illustrating fold change in expression of mRNA of genes *Nlrp3*, *Casp1*, *Il-1*β, and *Il-18*, *Asc*, *Il-10*, and *Tnf* in DIO, hyperleptinemia, and *ob/ob* models in comparison with respective controls, determined by real-time PCR. The scale on the right matches colors to log2 fold change (log2 FC) values. Data normalized to mRNA expression of ribosomal protein L37 (*Rpl37*). Abundance of **(C)** NLRP3, **(D)** pro CASP1 p45, **(E)** CASP1 p20, **(F)** pro IL-18 p24, and **(G)** IL-18 p18 in mouse liver of DIO, hyperleptinemic, and *ob/ob* mice measured in Western blotting analysis. Protein normalized with β-actin or glyceraldehyde 3-phosphate dehydrogenase (GAPDH) level. Bars represent mean ± SEM. Statistical analysis between groups was carried out using Mann–Whitney test. Number of samples: *n* = 6–9 for real-time PCR analysis and *n* = 4–8 for immunoblots. Asterisks indicate significant differences (*p < 0.05; **p < 0.01; +*p* = 0.07; +*p* = 0.08—all values indicated). AU, arbitrary units. **(H)** Diagram summarizing the profile of NLRP3 inflammasome activation in ovaries and liver of DIO mice. Figure 5H created with BioRender.com.

In the last experiment, we compared the temporal activation of NRLP3 inflammasome between the ovary and another metabolic organ as the liver, during obesity progression. Thus, we analyzed the expression profile of NLRP3 inflammasome genes in the liver of DIO mice. Furthermore, we tested once more the functional link between leptin signaling and activation of NLRP3 inflammasome in the liver, using pharmacological hyperleptinemic and *ob/ob* mouse models. Liver samples were collected from DIO, leptin treated, and *ob/ob* female mice, for mRNA transcription and protein expression analysis (Figure 5A). Real-time PCR analysis (*n* = 6–9/group) showed no significant changes in expression of all inflammasome components, except for the increase in *Nlrp3* and *Il-1*β mRNA in 16-week HFD, but downregulation of *Nlrp3* in *ob/ob*−/− (Figure 5B, *p* < 0.05). Furthermore, protein analysis determined by Western blotting (*n* = 4–8/group) showed an increase in protein levels of NLRP3, CASP1 (p20), and IL-18 (p18) after 16-week HFD treatment, compared with control (Figures 5C,E,G, *p* < 0.05, *p* = 0.07, *p* = 0.08). Regarding the pro-peptide of CASP1 (p45), its protein was upregulated in 16 L, but downregulated in *ob/ob*−/−, compared with controls (Figure 5D, *p* < 0.05 and *p* < 0.01, respectively). Finally, CASP1 (p20) and IL-18 (p18) proteins were decreased in *ob/ob*−/− (Figures 5E,G, *p* < 0.05 and *p* = 0.08, respectively). No significant changes were observed for pro IL-18 (p24) (Figure 5F). The present results of our analysis in the liver indicate a site-dependent NLRP3 inflammasome regulation throughout obesity, since overexpression of NLRP3, CASP1 (p20), and IL-18 (p18) took place only at 16 week of DIO. Thus, we observed drastic differences in NLRP3 inflammasome profile between liver and ovary (Figure 5H), which showed the upregulation of NLRP inflammasome components after 4-week DIO (Figure 2). Another important observation was the downregulation of NLRP3 and CASP1 (p20) in livers from *ob/ob* −/− mice, suggesting that leptin directly modulates NLRP3 inflammasome activation at hepatic level. Hence, we confirmed the latency of NLRP3 inflammasome activation in the liver of DIO female mice, which showed signs of upregulation only after 16- week HFD treatment. Furthermore, we have confirmed the functional link between leptin and NLRP3 inflammasome activation in the liver.

## Discussion

The present study gives the first characterization of NLRP3-induced inflammasome activation in the ovaries of DIO mice. Maternal obesity has been largely associated with increased ovarian inflammation (Robker et al., 2011; Nteeba et al., 2013, 2014; Ruebel et al., 2017; Snider and Wood, 2019). Therefore, a better knowledge of its pathogenesis will contribute to our understanding of the mechanisms underpinning ovarian failure and infertility during obesity. We first studied the effects of cyclicity on NLRP3 inflammasome activation in the ovaries of lean mice, confirming the upregulation of NLRP3 inflammasome components during D. Subsequently, we characterized the expression profile of NLRP3 inflammasome components in the ovary throughout obesity progression. Indeed, the rapid upregulation of NLRP3 protein in early obesity (after 4- week HFD treatment) was followed by a consistent downregulation of NLRP3, IL-18, and CASP1 in late obesity (after 16- week HFD). Importantly, using either a pharmacological hyperleptinemic and a genetic obese *ob/ob* mice, we evidenced the functional link between levels of leptin signaling and NLRP3 activation in whole ovaries. Furthermore, in CCs collected after superovulation, we confirmed also the major role of leptin on the upregulation of *Nlrp3*, *Casp1*, *Asc*, *Il-18*, and *Il-1*β transcription. Finally, after analyzing the NLRP3 inflammasome expression pattern in the liver, we observed NLRP3, CASP1, and IL-18 upregulation exclusively after 16- week HFD treatment, showing a delayed activation of NLRP3 inflammasome activation with regard to the ovary. Hence, these results suggest a greater vulnerability of the ovaries, in general, and the gamete, in particular, to the energetic surplus that females face under obesogenic conditions.

A recent study by Zhang and colleagues showed for the first time the expression of NLRP3 protein in various cellular components of mouse ovaries, like GC, TC, and oocytes (Zhang et al., 2019). We presently observed not only a similar pattern of cellular expression for NLRP3 but also the upregulation of NLRP3 inflammasome components during D. These findings corroborate previous results from Zhang and colleagues suggesting the involvement of NLRP3 in inflammatory events during ovulation in mice (Zhang et al., 2019). Moreover, NLRP3 was recently characterized as an important mediator of ovarian pathology during aging (Navarro-Pando et al., 2021). Finally, the involvement of NLRP3 inflammasome in ovarian inflammatory response in obese mothers is supported by previous reports showing NLRP3 inflammasome activation in proinflammatory conditions like PCOS (Wang et al., 2017; Guo et al., 2020). Thus, NLRP3 inflammasome may contribute for the regulation of key mechanisms during ovarian physiology and pathology.

The NLRP3 inflammasome is a critical component of innate immunity, frequently associated with human disease (He et al., 2016a). Our results evidencing NLRP3 inflammasome activation in the ovaries in early obesity (after 4- week HFD) are in line with previous reports showing the accumulation of proinflammatory mediators in the ovary of mice after short-term (6- week HFD) DIO (Shen et al., 2021). Indeed, we presently observed the upregulation of IL-18 protein, as well as the mRNA of *Tnf* (data not shown), after 4- week HFD supporting the early establishment of inflammatory response in the ovaries of DIO mice. Nonetheless, the regulation of ovarian inflammation in late obesity (16- week HFD) seems to be independent from NLRP3 inflammasome activation, as IL-18 and CASP1 mature proteins were significantly downregulated. Undeniably, increased levels of IL-1β in the ovaries of 16- week HFD mice confirmed the proinflammatory state existent in the ovaries. Of note, our results were confirmed by another study showing the upregulation of the proinflammatory mediators like *Il-1*β, *Il-6*, and *Tnf*α in the ovaries of mice fed HFD for 24 week (Nteeba et al., 2013). Hence, our results suggest the existence of alternative pathways to NLRP3 inflammasome mediating IL-1β upregulation in mouse ovaries at 16- week HFD (Schmidt and Lenz, 2012; Zhu and Kanneganti, 2017; Donado et al., 2020; Jain et al., 2020; Pyrillou et al., 2020).

We have recently characterized the temporal pattern of expression of proinflammatory genes in CCs from DIO mice (Wołodko et al., 2020). In early obesity, inflammatory cues in CCs were mediated by the upregulation of genes involved in cellular response to stress as *DEAD-box helicase 5 (Ddx5)*, *hypoxia inducible factor 1 subunit alpha (Hif1a)*, and *ADAM metallopeptidase domain 9 (Adam9*). Indeed, mediators of stress response like reactive oxygen species are known to prime the NLRP3 inflammasome (Gurung et al., 2014). Subsequently, in late obesity, we observed the overexpression of genes involved in anatomical structural morphogenesis, such as *C–C motif chemokine ligand 7* (*Ccl7*), an important chemoattractant of leukocytes (Menten et al., 1999). Furthermore, *Ccl7* is also known to interact with matrix metalloproteinases (MMPs) (Liu et al., 2018) or complement C3a receptor 1 (*C3ar1*), a component of the complement known to mediate neutrophil mobilization (Brennan et al., 2019) and lately described as a marker of PCOS progression (He et al., 2020). Therefore, these data suggest important temporal changes in the regulation of the inflammatory response in the ovary of mice throughout obesity progression. Indeed, NLRP3 inflammasome seems to play a critical role mostly in the initiation of inflammation in the ovaries in early obesity. Conversely, in late obesity, immune-mediated response in the ovary mainly comprises the infiltration of immune cells and mediation of structural reorganization, independent from the activation of NLRP3 inflammasome.

Our study also sheds light on the important crosstalk between leptin signaling and inflammasome NLRP3 activation in the ovary of DIO mice. As reviewed by Wani et al. (2021), numerous factors were shown to activate NLRP3 inflammasome during obesity, such as cellular metabolites, carbohydrates, or lipids. Nonetheless, leptin, a conserved proinflammatory cytokine (Iikuni et al., 2008; La Cava, 2017), was recently shown to upregulate NLRP3 components *in vitro* (Fu et al., 2017). Thus, in order to test the hypothesis whether repression of NLRP3 inflammasome activation in ovaries of 16- week HFD mice was associated with the local establishment of leptin resistance (Wołodko et al., 2020), we studied NLRP3 inflammasome activation in the ovaries of pharmacological hyperleptinemic and *ob/ob* mice. Strikingly, we observed a consistent upregulation of NLRP3 inflammasome components and accumulation of IL-1β protein in ovaries of leptin-treated mice. Conversely, in *ob/ob* −/− mice, NLRP3 and IL-1β proteins were downregulated. Furthermore, reduced levels of NLRP3 were also observed in *ob/ob* −/− mouse peritoneal macrophages treated with LPS and nigericin in comparison with wild-type mice (Yang et al., 2021). This certainly underlines the preponderant role of leptin on NLRP3 inflammasome regulation. Therefore, our results invite us to suggest an important link between levels of leptin signaling in the ovary of DIO mice and activation of NLRP3 inflammasome. In early obesity (4- week HFD), leptin actively signals through receptor b (ObRb) in the ovary (Wołodko et al., 2020), determining the overexpression of NLRP3 inflammasome components. Nonetheless, in late obesity (16- week HFD), and after the establishment of leptin resistance in the organ (Wołodko et al., 2020), the expression of NLRP3 inflammasome components is repressed.

Next, we reanalyzed our datasets on global gene expression in CCs collected from DIO and pharmacological hyperleptinemic mice in order to test the association between leptin signaling and activation of NLRP3 inflammasome in the somatic companions of the oocyte. Importantly, CCs are known as faithful indicators of intrafollicular environment (Wołodko et al., 2021), and their transcriptome has been previously used to predict oocyte and embryo quality (Uyar et al., 2013). Despite no changes in 4- week HFD, we confirmed the overexpression of NLRP3 inflammasome genes in CCs from 16 L. Consequently, we interrogated whether DEGs overlapping both 16 L and 4- week HFD treatment could interact with NLRP3 inflammasome genes. Indeed, gene ontology for associated genes revealed key terms for oocyte maturation, such as regulation of glucose transport, response to ATP, and regulation of cytokine biosynthesis. Metabolic regulation in preovulatory follicles appears to control major steps for maturation of female gamete, such as meiosis resumption, chromatin condensation, and cytoplasm maturation (Wołodko et al., 2021). For instance, glucose metabolism, which takes place mostly in CCs (Sanfins et al., 2018) was shown to be a key for oocyte competence (Wilding et al., 2009). Furthermore, reduced ATP content in oocytes was linked to failure in fertilization, arrested division, and abnormal embryonic development (Zhao and Li, 2012). Furthermore, NLRP3 inflammasome was previously reported to be involved in ovulation (Zhang et al., 2019). Given both direct and indirect roles of leptin in ovulation (Wołodko et al., 2021), the failure in leptin signaling and NLRP3 inflammasome activation in late obesity could account for increased anovulatory rates in obese mothers (Wu et al., 2010; Hou et al., 2016). Finally, the absence of changes in 4- week HFD in NLRP3 inflammasome genes can be ascribed to the low levels of coverage of our reduced-cell libraries and also the low levels of *Nlrp3* mRNA expression. Indeed, the present RNA-seq protocol used as little as 50 cells per mouse, which limits the analysis of weakly expressed genes. Collectively, our results indicate that leptin and NLRP3 inflammasome crosstalk in CCs of DIO mice can interfere with key steps for oocyte maturation, ovulation, and ultimately embryo development.

In the last experiment, we confirmed that the liver, in sharp contrast to the ovary, activated NLRP3 inflammasome later in time during DIO (after 16- week HFD treatment). Temporal differences in the regulation of inflammatory response (Figure 5H) certainly rely on the contrasting exposition to exogenous pathogens both organs face. The liver is constantly exposed to proinflammatory mediators from dietary and commensal bacterial products (Robinson et al., 2016). Thus, the hepatic immune system is constantly in contact with altered metabolic activity and regular exposition to microbial products, which results in persistent and tightly regulated inflammatory response (Robinson et al., 2016). On the contrary, the ovary is not only a highly immunogenic organ constantly secreting large amounts of cytokines and immune mediators (Piccinni et al., 2021) but also more prone to rapidly mount a proinflammatory response during obesity. Indeed, the inability of the ovary to control inflammation, and the exacerbated activation of cytokine production, certainly ascribes for the great vulnerability of the female gamete to maternal obesity even at earlier stages of disease progression. Thus, our results expose the increased vulnerability of the ovaries to maternal obesity, characterized by the development of an early inflammatory response that rapidly affects the gamete and impairs fertilization.

In summary, our work evidences the major role of leptin signaling on NLRP3 inflammasome activation in the ovary of mice during early obesity. Noteworthy, failure in ovarian leptin signaling in late obesity was associated with the repression in NLRP3 activity, but with maintenance of inflammation and levels of IL-1β. Moreover, NLRP3 inflammasome activation in the ovaries after early obesity contrasted with its activation exclusively during late obesity in the liver. Hence, the present findings suggest a greater vulnerability of the ovary, in general, and the gamete, in particular, to the energetic surplus during maternal obesity.

## Data Availability Statement

The data presented in the study are deposited in the Gene Expression Omnibus repository, accession number GSE180300.

## Ethics Statement

The animal study was reviewed and approved by the Local Animal Care and Use Committee for University of Warmia and Mazury, Olsztyn. Guidelines for animal experiments followed EU Directive 2010/63/EU.

## Author Contributions

MA acquired, analyzed, interpreted the data, and wrote the manuscript. KW acquired, analyzed the data, revised, and edited the manuscript. JO performed the immunohistochemistry staining. JC-F conducted the data analysis and interpretation of data and revised the manuscript. DM performed the immunohistochemistry staining. GK revised and edited the manuscript. AG conceptualized and designed the study, acquired the funding, participated in the acquisition, analysis, interpretation of the data, and wrote and edited the manuscript. All authors contributed to the article and approved the submitted version.

## Conflict of Interest

The authors declare that the research was conducted in the absence of any commercial or financial relationships that could be construed as a potential conflict of interest.

## Publisher’s Note

All claims expressed in this article are solely those of the authors and do not necessarily represent those of their affiliated organizations, or those of the publisher, the editors and the reviewers. Any product that may be evaluated in this article, or claim that may be made by its manufacturer, is not guaranteed or endorsed by the publisher.

## Footnotes

1
https://www.ncbi.nlm.nih.gov/geo/query/acc.cgi?acc=GSE180300

## References

[B1] Barlan A. U. Griffin T. M. McGuire K. A. Wiethoff C. M. (2011). Adenovirus membrane penetration activates the NLRP3 inflammasome. *J. Virol.* 85 146–155. 10.1128/JVI.01265-10 20980503 PMC3014182

[B2] Billon C. Murray M. H. Avdagic A. Burris T. P. (2019). RORγ regulates the NLRP3 inflammasome. *J. Biol. Chem.* 294 10–19. 10.1074/jbc.AC118.002127 30455347 PMC6322869

[B3] Blüher M. (2019). Obesity: global epidemiology and pathogenesis. *Nat. Rev. Endocrinol.* 15 288–298. 10.1038/s41574-019-0176-8 30814686

[B4] Boden G. (2002). Interaction between free fatty acids and glucose metabolism. *Curr. Opin. Clin. Nutr. Metab. Care* 5 545–549. 10.1097/00075197-200209000-00014 12172479

[B5] Brennan F. H. Jogia T. Gillespie E. R. Blomster L. V. Li X. X. Nowlan B. (2019). Complement receptor C3aR1 controls neutrophil mobilization following spinal cord injury through physiological antagonism of CXCR2. *JCI Insight* 4:e98254. 10.1172/jci.insight.98254 31045582 PMC6538362

[B6] Cauble R. Hamad S. Herring E. Lichtenwalter C. McGuire C. Dridi S. (2018). Leptin activates NLRP3 inflammasome-associated with Type II diabetes and obesity. *Adv. Food Technol. Nutr. Sci. Open J.* 4 e13–e16. 10.17140/AFTNSOJ-4-e016

[B7] Chaves de Souza J. A. Nogueira A. V. B. Chaves de Souza P. P. Kim Y. J. Lobo C. S. Pimentel Lopes de Oliveira G. J. (2013). SOCS3 expression correlates with severity of inflammation, expression of proinflammatory cytokines, and activation of STAT3 and P38 MAPK in LPS-induced inflammation in vivo. *Mediators Inflamm.* 2013:650812. 10.1155/2013/650812 24078776 PMC3775441

[B8] Chu S. Y. Kim S. Y. Lau J. Schmid C. H. Dietz P. M. Callaghan W. M. (2007). Maternal obesity and risk of stillbirth: a metaanalysis. *Am. J. Obstet. Gynecol.* 197 223–228. 10.1016/j.ajog.2007.03.027 17826400

[B9] Csárdi G. Nepusz T. (2006). The igraph software package for complex network research. *InterJournal Complex Syst.* 1695:1695.

[B10] Czech M. P. (2020). Mechanisms of insulin resistance related to white, beige, and brown adipocytes. *Mol. Metab.* 34 27–42. 10.1016/j.molmet.2019.12.014 32180558 PMC6997501

[B11] Davis B. K. Wen H. Ting J. P.-Y. (2011). The inflammasome NLRs in immunity, inflammation, and associated diseases. *Annu. Rev. Immunol.* 29 707–735. 10.1146/annurev-immunol-031210-101405 21219188 PMC4067317

[B12] De Nardo D. Latz E. (2011). NLRP3 inflammasomes link inflammation and metabolic disease. *Trends Immunol.* 32 373–379. 10.1016/j.it.2011.05.004 21733753 PMC3151541

[B13] Donado C. A. Cao A. B. Simmons D. P. Croker B. A. Brennan P. J. Brenner M. B. (2020). A two-cell model for IL-1β release mediated by death-receptor signaling. *Cell Rep.* 31:107466. 10.1016/j.celrep.2020.03.030 32268091 PMC7192215

[B14] Fu S. Liu L. Han L. Yu Y. (2017). Leptin promotes IL-18 secretion by activating the NLRP3 inflammasome in RAW 264.7 cells. *Mol. Med. Rep.* 16 9770–9776. 10.3892/mmr.2017.7797 29039567

[B15] Galvão A. Henriques S. Pestka D. Lukasik K. Skarzynski D. Maria Mateus L. (2012). Equine luteal function regulation may depend on the interaction between cytokines and vascular endothelial growth factor: an in vitro study. *Biol. Reprod.* 86:187. 10.1095/biolreprod.111.097147 22492973

[B16] Galvão A. Tramontano A. Rebordão M. R. Amaral A. Bravo P. P. Szóstek A. (2014). Opposing roles of leptin and ghrelin in the equine Corpus luteum regulation: an in vitro study. *Mediators Inflamm.* 2014:e682193. 10.1155/2014/682193 25125800 PMC4122068

[B17] Guglielmo A. Sabra A. Elbery M. Cerveira M. M. Ghenov F. Sunasee R. (2017). A mechanistic insight into curcumin modulation of the IL-1β secretion and NLRP3 S-glutathionylation induced by needle-like cationic cellulose nanocrystals in myeloid cells. *Chem. Biol. Interact.* 274 1–12. 10.1016/j.cbi.2017.06.028 28669709

[B18] Guo Q.-J. Shan J. Xu Y.-F. Hu Y.-Y. Huo C.-L. Song J.-Y. (2020). Pioglitazone metformin complex improves polycystic ovary syndrome comorbid psychological distress via inhibiting NLRP3 inflammasome activation: a prospective clinical study. *Mediators Inflamm.* 2020:3050487. 10.1155/2020/3050487 32410849 PMC7204303

[B19] Gurung P. Anand P. K. Malireddi R. K. Vande Walle L. Van Opdenbosch N. Dillon C. P. (2014). FADD and caspase-8 mediate priming and activation of the canonical and noncanonical Nlrp3 inflammasomes. *J. Immunol. (Baltimore, Md. 1950)* 192 1835–1846. 10.4049/jimmunol.1302839 24453255 PMC3933570

[B20] Hasegawa A. Mochida K. Inoue H. Noda Y. Endo T. Watanabe G. (2016). High-Yield superovulation in adult mice by anti-inhibin serum treatment combined with estrous cycle synchronization. *Biol. Reprod.* 94:21. 10.1095/biolreprod.115.134023 26632610

[B21] He D. Liu L. Wang Y. Sheng M. (2020). A novel genes signature associated with the progression of polycystic ovary syndrome. *Pathol. Oncol. Res. POR* 26 575–582. 10.1007/s12253-019-00676-3 31278444

[B22] He Y. Zeng M. Y. Yang D. Motro B. Núñez G. (2016b). NEK7 is an essential mediator of NLRP3 activation downstream of potassium efflux. *Nature* 530 354–357. 10.1038/nature16959 26814970 PMC4810788

[B23] He Y. Hara H. Núñez G. (2016a). Mechanism and regulation of NLRP3 inflammasome activation. *Trends Biochem. Sci.* 41 1012–1021. 10.1016/j.tibs.2016.09.002 27669650 PMC5123939

[B24] Hotamisligil G. S. Erbay E. (2008). Nutrient sensing and inflammation in metabolic diseases. *Nat. Rev. Immunol.* 8 923–934. 10.1038/nri2449 19029988 PMC2814543

[B25] Hou Y.-J. Zhu C.-C. Duan X. Liu H.-L. Wang Q. Sun S.-C. (2016). Both diet and gene mutation induced obesity affect oocyte quality in mice. *Sci. Rep.* 6:18858. 10.1038/srep18858 26732298 PMC4702149

[B26] Hughes M. M. O’Neill L. A. J. (2018). Metabolic regulation of NLRP3. *Immunol. Rev.* 281 88–98. 10.1111/imr.12608 29247992

[B27] Hughes M. M. Hooftman A. Angiari S. Tummala P. Zaslona Z. Runtsch M. C. (2019). Glutathione transferase omega-1 regulates NLRP3 inflammasome activation through NEK7 deglutathionylation. *Cell Rep.* 29 151–161.e5. 10.1016/j.celrep.2019.08.072 31577945

[B28] Iikuni N. Lam Q. L. K. Lu L. Matarese G. La Cava A. (2008). Leptin and inflammation. *Curr. Immunol. Rev.* 4 70–79. 10.2174/157339508784325046 20198122 PMC2829991

[B29] Iyer S. S. He Q. Janczy J. R. Elliott E. I. Zhong Z. Olivier A. K. (2013). Mitochondrial cardiolipin is required for Nlrp3 inflammasome activation. *Immunity* 39 311–323. 10.1016/j.immuni.2013.08.001 23954133 PMC3779285

[B30] Jain A. Irizarry-Caro R. A. McDaniel M. M. Chawla A. S. Carroll K. R. Overcast G. R. (2020). T cells instruct myeloid cells to produce inflammasome-independent IL-1β and cause autoimmunity. *Nat. Immunol.* 21 65–74. 10.1038/s41590-019-0559-y 31848486 PMC6927526

[B31] Jo E.-K. Kim J. K. Shin D.-M. Sasakawa C. (2016). Molecular mechanisms regulating NLRP3 inflammasome activation. *Cell. Mol. Immunol.* 13 148–159. 10.1038/cmi.2015.95 26549800 PMC4786634

[B32] Jungheim E. S. Schoeller E. L. Marquard K. L. Louden E. D. Schaffer J. E. Moley K. H. (2010). Diet-Induced obesity model: abnormal oocytes and persistent growth abnormalities in the offspring. *Endocrinology* 151 4039–4046. 10.1210/en.2010-0098 20573727 PMC2940512

[B33] Karlsson C. Lindell K. Svensson E. Bergh C. Lind P. Billig H. (1997). Expression of functional leptin receptors in the human ovary. *J. Clin. Endocrinol. Metab.* 82 4144–4148. 10.1210/jcem.82.12.4446 9398729

[B34] Kim S. R. Lee Y. C. Kim H. J. Kim S. H. (2015). Activation of NLRP3 inflammasome is regulated by mitochondrial ROS via PI3K-HIF-VEGF pathway in acute lung injury. *Eur. Respir. J.* 46(Suppl. 59):A3026. 10.1183/13993003.congress-2015.PA3026

[B35] Krebs M. Roden M. (2005). Molecular mechanisms of lipid-induced insulin resistance in muscle, Liver and Vasculature. *Diabetes Obes. Metab.* 7 621–632. 10.1111/j.1463-1326.2004.00439.x 16219006

[B36] Kyrönlahti A. Vetter M. Euler R. Bielinska M. Jay P. Y. Anttonen M. (2011). GATA4 deficiency impairs ovarian function in adult mice. *Biol. Reprod.* 84 1033–1044. 10.1095/biolreprod.110.086850 21248289 PMC3080425

[B37] La Cava A. (2017). Leptin in inflammation and autoimmunity. *Cytokine* 98 51–58. 10.1016/j.cyto.2016.10.011 27916613 PMC5453851

[B38] Lamkanfi M. (2011). Emerging inflammasome effector mechanisms. *Nat. Rev. Immunol.* 11 213–220. 10.1038/nri2936 21350580

[B39] Lamkanfi M. Dixit V. M. (2014). Mechanisms and functions of inflammasomes. *Cell* 157 1013–1022. 10.1016/j.cell.2014.04.007 24855941

[B40] Li Y. Zheng J.-Y. Liu J.-Q. Yang J. Liu Y. Wang C. (2016). Succinate/NLRP3 inflammasome induces synovial fibroblast activation: therapeutical effects of clematichinenoside AR on arthritis. *Front. Immunol.* 7:532. 10.3389/fimmu.2016.00532 28003810 PMC5141240

[B41] Liu Y. Cai Y. Liu L. Wu Y. Xiong X. (2018). Crucial biological functions of CCL7 in cancer. *PeerJ* 6:e4928. 10.7717/peerj.4928 29915688 PMC6004300

[B42] Loche E. Blackmore H. L. Carpenter A. A. Beeson J. H. Pinnock A. Ashmore T. J. (2018). Maternal diet-induced obesity programmes cardiac dysfunction in male mice independently of post-weaning diet. *Cardiovasc. Res.* 114 1372–1384. 10.1093/cvr/cvy082 29635288 PMC6054211

[B43] Martine P. Chevriaux A. Derangère V. Apetoh L. Garrido C. Ghiringhelli F. (2019). HSP70 Is a Negative regulator of NLRP3 inflammasome activation. *Cell Death Dis.* 10 1–11. 10.1038/s41419-019-1491-7 30874540 PMC6420651

[B44] Martinon F. Burns K. Tschopp J. (2002). The inflammasome: a molecular platform triggering activation of inflammatory caspases and processing of ProIL-beta. *Mol. Cell* 10 417–426. 10.1016/s1097-2765(02)00599-3 12191486

[B45] Martinon F. Pétrilli V. Mayor A. Tardivel A. Tschopp J. (2006). Gout-Associated uric acid crystals activate the NALP3 inflammasome. *Nature* 440 237–241. 10.1038/nature04516 16407889

[B46] Menten P. Proost P. Struyf S. Van Coillie E. Put W. Lenaerts J. P. (1999). Differential induction of monocyte chemotactic protein-3 in mononuclear leukocytes and fibroblasts by interferon-alpha/beta and interferon-gamma reveals MCP-3 heterogeneity. *Eur. J. Immunol.* 29 678–685. 10064085 10.1002/(SICI)1521-4141(199902)29:02<678::AID-IMMU678>3.0.CO;2-J

[B47] Minge C. E. Bennett B. D. Norman R. J. Robker R. L. (2008). Peroxisome proliferator-activated receptor-gamma agonist rosiglitazone reverses the adverse effects of diet-induced obesity on oocyte quality. *Endocrinology* 149 2646–2656. 10.1210/en.2007-1570 18276752

[B48] Mitoma H. Hanabuchi S. Kim T. Bao M. Zhang Z. Sugimoto N. (2013). The DEAH Box RNA helicase DHX33 senses cytosolic RNA and activates the NLRP3 inflammasome. *Immunity* 39 123–135. 10.1016/j.immuni.2013.07.001 23871209 PMC3756931

[B49] Navarro-Pando J. M. Alcocer-Gómez E. Castejón-Vega B. Navarro-Villarán E. Condés-Hervás M. Mundi-Roldan M. (2021). Inhibition of the NLRP3 inflammasome prevents ovarian aging. *Sci. Adv.* 7:eabc7409. 10.1126/sciadv.abc7409 33523841 PMC7775749

[B50] Nigam S. K. (2018). The SLC22 transporter family: a paradigm for the impact of drug transporters on metabolic pathways, signaling, and disease. *Annu. Rev. Pharmacol. Toxicol.* 58 663–687. 10.1146/annurev-pharmtox-010617-052713 29309257 PMC6225997

[B51] Nteeba J. Ganesan S. Keating A. F. (2014). Progressive obesity alters ovarian Folliculogenesis with impacts on pro-inflammatory and steroidogenic signaling in female mice. *Biol. Reprod.* 91:86. 10.1095/biolreprod.114.121343 25143355 PMC4435031

[B52] Nteeba J. Ortinau L. C. Perfield J. W. Keating A. F. (2013). Diet-Induced obesity alters immune cell infiltration and expression of inflammatory cytokine genes in mouse ovarian and peri-ovarian adipose depot tissues. *Mol. Reprod. Dev.* 80 948–958. 10.1002/mrd.22231 24038509

[B53] Odegaard J. I. Chawla A. (2008). Mechanisms of macrophage activation in obesity-induced insulin resistance. *Nat. Clin. Pract. Endocrinol. Metab.* 4 619–626. 10.1038/ncpendmet0976 18838972 PMC3381907

[B54] Palazón-Riquelme P. Worboys J. D. Green J. Valera A. Martín-Sánchez F. Pellegrini C. (2018). USP7 and USP47 deubiquitinases regulate NLRP3 inflammasome activation. *EMBO Rep.* 19:e44766. 10.15252/embr.201744766 30206189 PMC6172458

[B55] Penzias A. S. (2012). Recurrent IVF failure: other factors. *Fertil. Steril.* 97 1033–1038. 10.1016/j.fertnstert.2012.03.017 22464759

[B56] Piccinni M.-P. Vicenti R. Logiodice F. Fabbri R. Kullolli O. Pallecchi M. (2021). Description of the follicular fluid cytokine and hormone profiles in human physiological natural cycles. *J. Clin. Endocrinol. Metab.* 106 e721–e738. 10.1210/clinem/dgaa880 33247906 PMC7823236

[B57] Pyrillou K. Burzynski L. C. Clarke M. C. H. (2020). Alternative pathways of IL-1 activation, and its role in health and disease. *Front. Immunol.* 11:613170. 10.3389/fimmu.2020.613170 33391283 PMC7775495

[B58] Robinson M. W. Harmon C. O’Farrelly C. (2016). Liver immunology and its role in inflammation and homeostasis. *Cell. Mol. Immunol.* 13 267–276. 10.1038/cmi.2016.3 27063467 PMC4856809

[B59] Robker R. L. (2008). Evidence that obesity alters the quality of oocytes and embryos. *Pathophysiology* 15 115–121. 10.1016/j.pathophys.2008.04.004 18599275

[B60] Robker R. L. Wu L. L.-Y. Yang X. (2011). Inflammatory pathways linking obesity and ovarian dysfunction. *J. Reprod. Immunol. XI Int. Congr. Reprod. Immunol.* 88 142–148. 10.1016/j.jri.2011.01.008 21333359

[B61] Rostamtabar M. Esmaeilzadeh S. Karkhah A. Amiri M. Rahmani A. Bakouei F. (2020). Elevated expression of IL-18 but not IL-1β gene is associated with NALP3 and AIM2 inflammasome in polycystic ovary syndrome. *Gene* 731:144352. 10.1016/j.gene.2020.144352 31935500

[B62] Ruebel M. L. Cotter M. Sims C. R. Moutos D. M. Badger T. M. Cleves M. A. (2017). Obesity modulates inflammation and lipid metabolism oocyte gene expression: a single-cell transcriptome perspective. *J. Clin. Endocrinol. Metab.* 102 2029–2038. 10.1210/jc.2016-3524 28323970 PMC5470765

[B63] Ryan N. K. Woodhouse C. M. Van der Hoek K. H. Gilchrist R. B. Armstrong D. T. Norman R. J. (2002). Expression of leptin and its receptor in the murine ovary: possible role in the regulation of oocyte maturation. *Biol. Reprod.* 66 1548–1554. 10.1095/biolreprod66.5.1548 11967222

[B64] Samuelsson A.-M. Matthews P. A. Argenton M. Christie M. R. McConnell J. M. Jansen E. H. J. M. (2008). Diet-Induced obesity in female mice leads to offspring hyperphagia, adiposity, hypertension, and insulin resistance: a novel murine model of developmental programming. *Hypertension (Dallas Tex. 1979)* 51 383–392. 10.1161/HYPERTENSIONAHA.107.101477 18086952

[B65] Sanfins A. Rodrigues P. Albertini D. F. (2018). GDF-9 and BMP-15 direct the follicle symphony. *J. Assist. Reprod. Genet.* 35 1741–1750. 10.1007/s10815-018-1268-4 30039232 PMC6150895

[B66] Schäfer J. Strimmer K. (2005). A shrinkage approach to large-scale covariance matrix estimation and implications for functional genomics. *Stat. Appl. Genet. Mol. Biol.* 4:Article32. 10.2202/1544-6115.1175 16646851

[B67] Schmidt R. L. Lenz L. L. (2012). Distinct licensing of IL-18 and IL-1β secretion in response to NLRP3 inflammasome activation. *PLoS One* 7:e45186. 10.1371/journal.pone.0045186 23028835 PMC3445464

[B68] Shannon P. Markiel A. Ozier O. Baliga N. S. Wang J. T. Ramage D. (2003). Cytoscape: a software environment for integrated models of biomolecular interaction networks. *Genome Res.* 13 2498–2504. 10.1101/gr.1239303 14597658 PMC403769

[B69] Shao B.-Z. Xu Z.-Q. Han B.-Z. Su D.-F. Liu C. (2015). NLRP3 inflammasome and its inhibitors: a review. *Front. Pharmacol.* 6:262. 10.3389/fphar.2015.00262 26594174 PMC4633676

[B70] Shen H.-R. Xu X. Li X.-L. (2021). Berberine exerts a protective effect on rats with polycystic ovary syndrome by inhibiting the inflammatory response and cell apoptosis. *Reprod. Biol. Endocrinol.* 19:3. 10.1186/s12958-020-00684-y 33407557 PMC7789273

[B71] Shuvarikov A. A. Davis M. A. Esser-Nobis K. Gale M. J. (2018). Mitochondrial SLC25 proteins interact with NLRP3 to regulate inflammasome function. *J. Immunol.* 200(Suppl. 1):115.8.

[B72] Smith P. K. Krohn R. I. Hermanson G. T. Mallia A. K. Gartner F. H. Provenzano M. D. (1985). Measurement of protein using bicinchoninic acid. *Anal. Biochem.* 150 76–85. 10.1016/0003-2697(85)90442-7 3843705

[B73] Snider A. P. Wood J. R. (2019). Obesity induces ovarian inflammation and reduces oocyte quality. *Reproduction (Cambridge, England)* 158 R79–R90. 10.1530/REP-18-0583 30999278

[B74] Stienstra R. Tack C. J. Kanneganti T.-D. Joosten L. A. B. Netea M. G. (2012). The inflammasome puts obesity in the danger zone. *Cell Metab.* 15 10–18. 10.1016/j.cmet.2011.10.011 22225872

[B75] Tõzsér J. Benkõ S. (2016). Natural compounds as regulators of NLRP3 inflammasome-mediated IL-1β production. *Mediators Inflamm.* 2016:5460302. 10.1155/2016/5460302 27672241 PMC5031844

[B76] Traba J. Sack M. N. (2017). The role of caloric load and mitochondrial homeostasis in the regulation of the NLRP3 inflammasome. *Cell. Mol. Life Sci. CMLS* 74 1777–1791. 10.1007/s00018-016-2431-7 27942750 PMC5391300

[B77] Uyar A. Torrealday S. Seli E. (2013). Cumulus and Granulosa cell markers of oocyte and embryo quality. *Fertil. Steril.* 99 979–997. 10.1016/j.fertnstert.2013.01.129 23498999 PMC3866131

[B78] Wang F. Wang S. Zhang Z. Lin Q. Liu Y. Xiao Y. (2017). Activation of NLRP3 inflammasome in the ovaries during the development and treatment of polycystic ovary syndrome. *Int. J. Clin. Exp. Pathol.* 10 5022–5030.

[B79] Wang X. Jiang W. Yan Y. Gong T. Han J. Tian Z. (2014). RNA viruses promote activation of the NLRP3 inflammasome through a RIP1-RIP3-DRP1 signaling pathway. *Nat. Immunol.* 15 1126–1133. 10.1038/ni.3015 25326752

[B80] Wani K. AlHarthi H. Alghamdi A. Sabico S. Al-Daghri N. M. (2021). Role of NLRP3 inflammasome activation in obesity-mediated metabolic disorders. *Int. J. Environ. Res. Public Health* 18:511. 10.3390/ijerph18020511 33435142 PMC7826517

[B81] Weber A. N. R. Bittner Z. A. Shankar S. Liu X. Chang T.-H. Jin T. (2020). Recent insights into the regulatory networks of NLRP3 inflammasome activation. *J. Cell Sci.* 133:jcs248344. 10.1242/jcs.248344 33273068

[B82] Wen H. Gris D. Lei Y. Jha S. Zhang L. Huang M. T.-H. (2011). Fatty acid–induced NLRP3-ASC inflammasome activation interferes with insulin signaling. *Nat. Immunol.* 12 408–415. 10.1038/ni.2022 21478880 PMC4090391

[B83] Wilding M. Coppola G. Dale B. Di Matteo L. (2009). Mitochondria and human preimplantation embryo development. *Reproduction (Cambridge, England)* 137 619–624. 10.1530/REP-08-0444 19176592

[B84] Wolf A. J. Reyes C. N. Liang W. Becker C. Shimada K. Wheeler M. L. (2016). Hexokinase is an innate immune receptor for the detection of bacterial peptidoglycan. *Cell* 166 624–636. 10.1016/j.cell.2016.05.076 27374331 PMC5534359

[B85] Wołodko K. Castillo-Fernandez J. Kelsey G. Galvão A. (2021). Revisiting the impact of local leptin signaling in folliculogenesis and oocyte maturation in obese mothers. *Int. J. Mol. Sci.* 22:4270. 10.3390/ijms22084270 33924072 PMC8074257

[B86] Wołodko K. Walewska E. Adamowski M. Castillo-Fernandez J. Kelsey G. Galvão A. (2020). Leptin resistance in the ovary of obese mice is associated with profound changes in the transcriptome of cumulus cells. *Cell. Physiol. Biochem.* 54 417–437. 10.33594/000000228 32348667

[B87] Wu L. L.-Y. Dunning K. R. Yang X. Russell D. L. Lane M. Norman R. J. (2010). High-Fat diet causes lipotoxicity responses in cumulus–oocyte complexes and decreased fertilization rates. *Endocrinology* 151 5438–5445. 10.1210/en.2010-0551 20861227

[B88] Yamaguchi A. Hori O. Stern D. M. Hartmann E. Ogawa S. Tohyama M. (1999). Stress-Associated endoplasmic reticulum protein 1 (SERP1)/Ribosome-Associated membrane protein 4 (RAMP4) stabilizes membrane proteins during stress and facilitates subsequent glycosylation. *J. Cell Biol.* 147 1195–1204. 10.1083/jcb.147.6.1195 10601334 PMC2168098

[B89] Yang C.-C. Wu C.-H. Lin T.-C. Cheng Y.-N. Chang C.-S. Lee K.-T. (2021). Inhibitory effect of PPARγ on NLRP3 inflammasome activation. *Theranostics* 11 2424–2441. 10.7150/thno.46873 33500734 PMC7797672

[B90] Zhang T. Tsutsuki H. Islam W. Ono K. Takeda K. Akaike T. (2021). ATP exposure stimulates glutathione efflux as a necessary switch for NLRP3 inflammasome activation. *Redox Biol.* 41:101930. 10.1016/j.redox.2021.101930 33740502 PMC7995658

[B91] Zhang Z. Wang F. Zhang Y. (2019). Expression and contribution of NLRP3 inflammasome during the follicular development induced by PMSG. *Front. Cell Dev. Biol.* 7:256. 10.3389/fcell.2019.00256 31750302 PMC6842944

[B92] Zhao J. Li Y. (2012). Adenosine triphosphate content in human unfertilized oocytes, undivided zygotes and embryos unsuitable for transfer or cryopreservation. *J. Int. Med. Res.* 40 734–739. 10.1177/147323001204000238 22613437

[B93] Zhao S. Fernald R. D. (2005). Comprehensive algorithm for quantitative real-time polymerase chain reaction. *J. Comput. Biol. A J. Comput. Mol. Cell Biol.* 12 1047–1064. 10.1089/cmb.2005.12.1047 16241897 PMC2716216

[B94] Zhou R. Tardivel A. Thorens B. Choi I. Tschopp J. (2010). Thioredoxin-Interacting protein links oxidative stress to inflammasome activation. *Nat. Immunol.* 11 136–140. 10.1038/ni.1831 20023662

[B95] Zhu Q. Kanneganti T.-D. (2017). Cutting edge: distinct regulatory mechanisms control proinflammatory cytokines IL-18 and IL-1β. *J. Immunol.* 198 4210–4215. 10.4049/jimmunol.1700352 28468974 PMC5544497

